# Learning in radiation oncology: 12‐month experience with a new incident learning system

**DOI:** 10.1002/jmrs.823

**Published:** 2024-09-15

**Authors:** Krystle Crouch, Laura Adamson, Rachael Beldham‐Collins, Jonathan Sykes, David Thwaites

**Affiliations:** ^1^ Sydney West Radiation Oncology Network Sydney Australia; ^2^ Institute of Medical Physics, School of Physics University of Sydney Sydney Australia

**Keywords:** Incident reporting, quality and safety, radiation therapy, safety culture

## Abstract

**Introduction:**

Safety and quality improvement are essential to clinical practice in radiation therapy as planning and treatment increase in complexity and sophistication. An incident learning system (ILS) is a safety and quality improvement tool that can aid risk mitigation to improve patient safety and quality of care. The aim of this study was to quantify the impact of implementing a new e‐ILS, **L**earning **I**n **R**adiation **ON**cology (LIRON), on reporting and safety culture within a local health district (LHD).

**Methods:**

The ILS (LIRON) was implemented in 2020 with the intent of tracking actual incidents, near misses and procedural non‐compliances for analysis of root causes and contributing factors. A survey was conducted after 12 months of LIRON use, and distributed to radiation oncologists, radiation therapists and radiation oncology medical physicists within the LHD. Results were compared with the responses to a pre‐ILS implementation survey, to review changes in staff perceptions of safety culture, barriers to reporting and ILS understanding.

**Results:**

Survey response rates were similar at baseline and at the 12‐month follow‐up, 64% and 63%, respectively. Findings showed increased ILS participation (49–71%), increased perception of no barriers to reporting (34–43%) and increased encouragement to report (37–43%). Greater confidence in the department's ability to learn from the ILS was evident (24–46%).

**Conclusion:**

Initial findings of LIRON implementation show positive impact but warrant further long‐term review for greater understanding of its impact on staff perceptions, safety culture and improving departmental processes.

## Graphical abstract

Introduction of an incident learning incident (ILS) was shown to have had a positive impact on reporting and safety culture within a local health district (LHD). Survey findings correlate with existing literature that ILSs are effective tools for improving patient safety and departmental safety facilitating quality improvement.

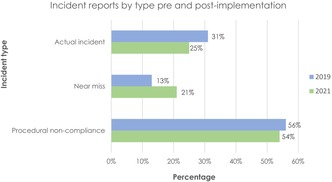

## Introduction

Radiation therapy is one of the main treatment options for cancer management with approximately 50% of Australian cancer patients benefiting from radiation therapy at some point in their cancer management plan.^1^ Treatment delivery is highly complex involving multiple interactions between members of the multidisciplinary team (MDT). The MDT includes radiation oncologists (ROs), radiation oncology medical physicists (ROMPs), radiation therapists (RTs), radiation oncology nurses, allied health and administration staff.

There are multiple processes and quality checkpoints throughout the patient workflow from the decision to treat, through to planning and treatment.^2^ The aim at each step is to ensure safe and accurate treatment is delivered for each fraction. The likelihood of a patient receiving a major dose deviation is rare due to robust quality assurance (QA) processes that have evolved in radiation therapy; however, the consequences of such error can be significant.^2^, ^3^

There is the potential for error at any point in the patient pathway; thus, current radiation oncology practice standards mandate all radiation oncology departments implement a safety and quality management (QSM) system for risk mitigation.^4^, ^5^, ^6^ Mandatory reporting in Australia occurs through state‐level bodies such as the New South Wales Environment Protection Authority (EPA) who then inform the Australian Radiation Protection and Nuclear Safety Agency (ARPANSA), as they are the caretaker of the Australian Radiation Incident Register (ARIR).^4^

A safety report from ARPANSA highlighted that errors submitted for mandatory reporting were most commonly the result of human error followed by equipment malfunction.^4^ Australian mandatory reporting is a requirement in radiation therapy for the following events^5^:

- Delivery of therapeutic radiation to the wrong patient;
- Delivery of therapeutic radiation to the wrong target volume; and
- Delivered dose that deviates by more than 10% from the prescribed treatment dose

### Incident reporting system

Incident learning systems (ILSs) are effective tools for improving patient safety and departmental safety culture (SC) while facilitating quality improvement. The process involves a feedback loop of error identification, reporting, detailed analysis, follow‐up, feedback and learning for all staff.^7^ Macrae et al. stated ‘We collect too much and do too little’ highlighting that incident reporting systems can fall short in providing recommendations for improving QA practices.^8^ A robust ILS should be more than simply logging incident reports of mandatory status; instead, it should include the whole learning process and can highlight systematic points of weakness or promote the development of new policies to prevent reoccurrence.^3^, ^9^

Incident learning in the radiation oncology context is challenging due to the need for additional training, resources and cultural changes. It is important to engage participation from staff at all levels for effective and efficient workflow.^10^, ^11^ No matter how developed an ILS is, successful implementation and uptake is dependent on departmental safety culture. Here, SC refers to the ‘individual and group beliefs, values, attitudes, perceptions, competencies and patterns of behaviour that determine the organisation's commitment to quality and patient safety’.^12^

SC is considered a just culture when the environment encourages staff to report any event without fear of negative consequences.^8^, ^9^ Various methods have been reported in the literature to establish an understanding of SC in radiation oncology. A number of these studies utilised surveying staff, primarily within a single department.^9^, ^10^, ^13^, ^14^, ^15^, ^16^, ^17^, ^18^, ^19^, ^20^, ^21^, ^22^

An investigation into baseline understanding of ILS and SC initiated the development of a radiation oncology specific electronic ILS called LIRON: Learning in Radiation ONcology.^9^, ^10^ The system went live at Western Sydney Local Health District (WSLHD) in December 2020. It is an electronic incident reporting system implemented with high accessibility to all staff via the Varian Aria™ oncology information system (Varian Medical Systems, Palo Alto, CA, USA).^9^

Staff are encouraged to report every incident or error including actual incidents (AI), near‐misses (NMI) and low‐level procedural non‐compliances (PNC) such as documentation errors that have missed detection at initial QA barriers. The MDT of RTs, ROMPs and ROs are provided training to correctly utilise the ILS and encouraged to use language that avoids blame and provides constructive recommendations.^9^, ^10^

LIRON is used alongside the mandatory, and more generalised NSW incident reporting system (IMS+). However, LIRON is tailored to radiation oncology incident reporting by having a number of workflow and functional advantages. All LIRON reports are tracked even when there is no clinical consequence to the patient, so causal factors and corrective actions can be identified to highlight any systematic QA weaknesses or educational needs.^10^

An incident triage team, with representation from all MDT members was established and trained to review submitted reports within 24 h. Reports are independently checked for accurate data input and correct data categorisation. Further investigation may include follow‐up with the initial reporter to promote root cause analysis and provide recommendations for leaders, working groups and quality improvement projects across all professional groups. Dissemination of safety information and feedback to the entire department is provided through newsletters, monthly LIRON MDT review and monthly radiation oncology quality assurance morbidity and mortality (QA M&M) meetings.

Actual incidents are undesirable events in pre‐treatment, planning or treatment that reach the patient and cause delivered treatment to deviate from the intended radiation therapy prescription.^3^, ^5^ A near miss incident is when a potential actual incident is prevented from reaching the patient through identification either by chance or at barrier points (e.g. QA checks) and is rectified prior to the delivery of erroneous treatment.^3^ A procedural non‐compliance is when a policy or procedure was not followed but did not have capacity for erroneous delivery or when multiple QA barriers fail to identify an error. Lower level incidents include near miss incidents and procedural non‐compliances.^7^, ^8^, ^9^

Procedural non‐compliance reporting expands a radiation oncology ILS's ability to learn from captured data due to the numerous QA checks involved pre‐treatment.^7^, ^10^, ^23^ A study by Frewen et al.^24^ found a patient can have up to 60 process steps and 141 sub‐process steps across the patient pathway. Thus, an ILS utilising low level procedural non‐compliance reporting has the capacity to understand any weak points in sub‐processes.

Multiple radiation oncology facilities have previously assessed the impact of developing or replacing their ILS with an electronic system, showing increased rates of reporting.^17^, ^18^, ^25^, ^26^, ^27^ Retrospective analysis of ILSs over time has shown a reduction in incident severity risk and in the number of incidents classified as actual incidents.^15^, ^28^, ^29^, ^30^, ^31^ These studies show an overall increase in reporting but decrease in risk categorisation and actual incidents. This suggests that including lower‐level reporting, such as the introduction of procedural non‐compliance reporting, strengthens QSM and QA pathways, showing a positive impact on risk mitigation. The reported decrease in actual incidents, and in severity and risk when incidents occur, indicate ILS implementation has a positive impact on patient safety in radiation oncology. Similar findings highlight the importance of including both NMI and AI for analysis, as causative factors and missed barrier points are often similar.^7^, ^14^, ^25^

The current literature describing the impact of an electronic ILS in the Australian radiation oncology context is limited.^10^, ^27^, ^30^, ^32^ This study aimed to quantify the impact of LIRON on the understanding and use of the ILS on safety and culture, to identify if it was consistent with published findings from other countries. A survey was used to assess the impact of the first 12‐months of LIRON utilisation, with comparison to a pre‐implementation survey previously used to establish a baseline level.^9^

## Methods and Materials

The project received ethics approval from the Western Sydney Local Health District (WSLHD) Human Research Ethics committee and Scientific Advisory QA committee.

### Incident learning system and safety culture survey tool

Prior to LIRON implementation in 2019, a departmental baseline survey was conducted to determine initial staff perceptions and attitudes towards ILSs and SC.^9^ A repeat anonymous online survey was distributed after 12‐months of LIRON use to evaluate its impact on incident reporting and safety culture within the department. The initial baseline survey included multiple cancer centres, while the repeat follow‐up survey was limited to two integrated cancer centres within a single LHD.

The follow‐up survey, consistent with the original one, contained 22 questions with an expected average completion time of around 10 min. The 22 survey questions were 16 multichoice using a 5‐point Likert scale, one free text answer question and two demographic questions (Appendix 1).

Participants were given the opportunity to provide further comments and feedback in free text at the conclusion of the survey. The survey content was the same as the baseline with minor changes such as the addition of ‘sometimes’ in the multichoice for several questions. Multiple staff stated ‘sometimes’ in open text at the baseline survey.

Microsoft Forms™ was used to host, capture and analyse results. A link to the e‐survey was distributed by email from a ‘gatekeeper’ RT to all ROs, ROMPs and RTs within the LHD. The 12‐month post‐implementation survey was open for 4 weeks with reminder emails sent weekly to provide updated response rates per cohort. Participants were informed that consent would be considered as having been provided upon acceptance of completing the survey.

### Data analysis

Results were exported to Excel for descriptive analysis of quantitative data. Baseline survey results concerning centres outside the local health district were excluded for comparative analysis.

## Results

### Characteristics of respondents

The 12‐month post‐implementation survey was distributed to 103 radiation oncology staff within WSLHD. The response rate was 63% (*n* = 65) compared to 64% (*n* = 70) at the pre‐ILS implementation survey. Breakdown of the 65 respondents by professional cohort were RTs 68% (*n* = 44), ROMPs 18% (*n* = 12) and ROs 14% (*n* = 9). By cohort, response rates were RT 66%, ROMP 100% and RO 38%. Participants reported a range of experience levels from recent graduates to experienced practitioners of more than 20 years.

### Characteristics of incident reports

**Figure 1:**
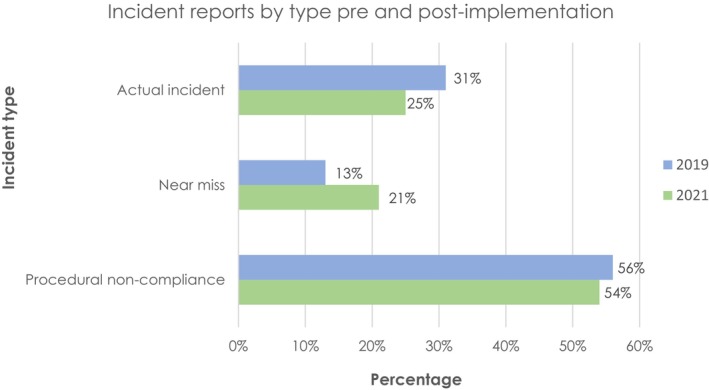
Submitted incident reports by type pre‐ (*n* = 298) and post‐LIRON implementation (*n* = 361).

Over a 12‐month period from September 2020 to September 2021, a total of 361 reports were submitted to the LIRON ILS. Representing a rate of 164 reports per 1000 treatment courses. Compared to 298 reports (134 reports per 1000 treatment courses submitted 12 months prior to LIRON implementation using the previous ‘deviation’ paper‐based reporting system). This highlights an increase in reporting with the electronic LIRON ILS. A summary of reported incidents by type is given in Figure 1.

Post‐LIRON implementation, the highest proportion of incidents was found to have originated at the treatment stage. A contributing factor for the increased number of actual incidents post‐LIRON could be attributed to the training and education provided when implementing LIRON as staff had a stronger awareness and understanding of correctly categorising reports supported by flowchart resources. For example, reporting repeat imaging due to human error (eg. incorrect setup instructions resulting in re‐setup and re‐imaging) was not historically classified as a documentation error. However, staff now report these under the category of procedural non‐compliance (repeated imaging) with a root cause of documentation.

**Figure 2:**
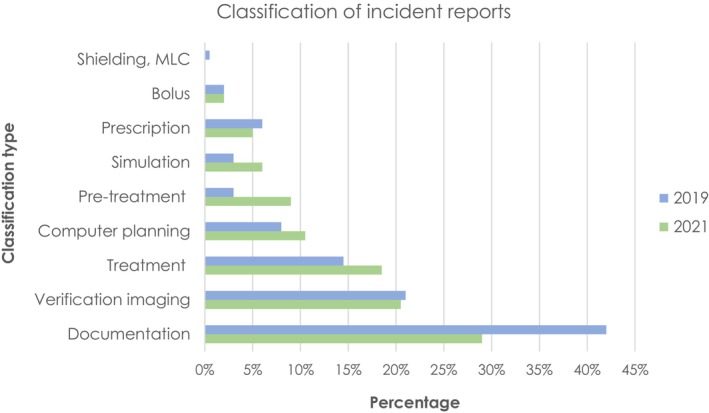
Incident reports by subcategory in 2019 (blue) and 2021 (green).

A breakdown of submitted reports by classification type is provided in Figure 2. Incorrect or missing documentation accounted for the highest proportion of incidents followed by pre‐treatment verification imaging and treatment related factors.

### Knowledge of incident reporting systems

Overall, awareness of an incident reporting system within the department increased from 97% at baseline to 100% at follow‐up. In 2021, 71% of respondents indicated they had submitted an actual, near miss or procedural non‐compliance in the previous 12 months, which demonstrated a 22% increase in staff reporting activity compared to 2019. Consistent with the old reporting system, incident submissions were predominantly completed by RTs (2019, 92%; 2021, 84%), followed by ROMPs (2019, 8%; 2021, 7%) and ROs (2019, 0%; 2021, 9%). These findings suggest increased reporting from ROs has occurred with the new MDT‐collaborated ILS and could be attributed to education initiatives and an RO representative at monthly QA meetings.

For the remaining 29% of respondents who did not report, their reasoning was due to not identifying anything to report, escalating to a team leader or asking another staff member to complete. In the 2019 baseline survey, an individual chose not to report, while several participants acknowledged near misses that would have been reported if the local system was clearer on where and what to report. Positively, responses indicating missed reporting were not observed post‐implementation.

**Figure 3:**
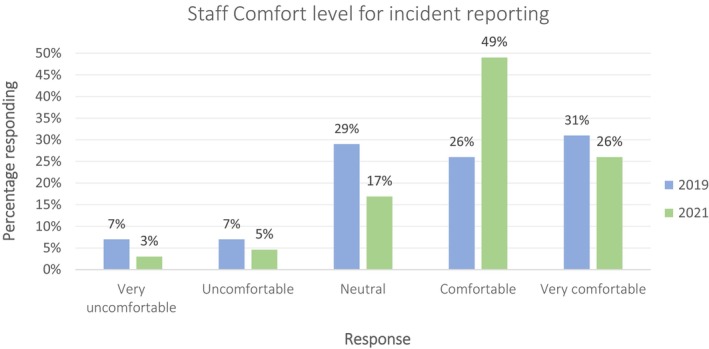
Staff‐reported comfort level for incident reporting in 2019 (blue) and 2021 (green).

The percentage of respondents who reported being comfortable with incident reporting increased significantly from 26% to 49% (pre‐ vs. post‐ILS; Fig. 3) and comfortable or very comfortable from 57% to 75%. Overall, this suggests greater staff confidence in reporting and use of the ILS.

### Utilisation and barriers to reporting

**Figure 4:**
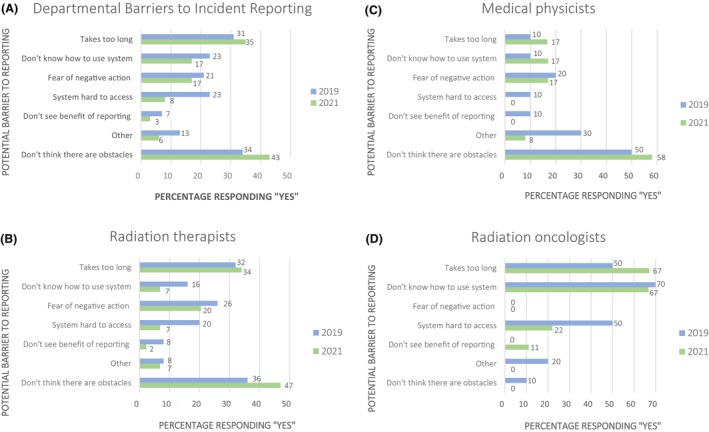
Self‐reported perceived barriers to incident reporting in 2019 (blue) and 2021 (green) for the whole staff cohort and by each professional cohort.

An increase was observed in the number of participants who believed there were no barriers to incident reporting (2019, 34%; 2021, 43%). A decline in nearly all perceived barriers was noted (Fig. 4A). However, respondents consistently acknowledged their biggest obstacle to reporting was that it takes too long (2019, 31%; 2021, 35%). RTs showed an improved understanding of the ILS system and decreased fear of punitive action (Fig. 4B). ROMPs demonstrated an improved perception of ILS accessibility and benefits of reporting (Fig. 4C). For ROs, improvement in understanding of the ILS was noted (Fig. 4D). These results highlight the most noted barriers were related to the reporting system rather than departmental safety culture.

### Safety culture

Post‐ILS implementation, the number of staff that felt encouraged to report slightly increased (2019, 67%; 2021, 70%) with most staff believing the department practiced a no‐blame culture (2019, 83%; 2021, 92%). Compared with other cohorts, ROMPs noted the biggest improvement in perception of a departmental no‐blame culture (2019, 50%; 2021, 83%).

Regarding staff concerns related to blame and punitive action (impacting on perceptions of a no‐blame culture), an increased proportion of staff (2019, 20%; 2021, 23%) noted that they had previously witnessed or personally received negative action due to incident reporting, which warrants further investigation. However, considering the classification of cause and blame between staff or system processes, this shifted to considering more blame to be on systems than on staff post implementation than was perceived pre‐ILS (Table 1).

**Table 1:** Classification of cause and blame due to incident reporting in 2019 and 2021.

Response	2019		2021	
	*n*	%	*n*	%
0% on staff, 100% on system	2	3%	4	6%
25% on staff, 75% on system	21	30%	26	40%
50% on staff, 50% on system	28	40%	18	28%
75% on staff, 25% on system	12	17%	7	11%
100% on staff, 0% on system	0	0%	0	0%
Other	7	10%	9	14%

### Feedback and learning

**Figure 5:**
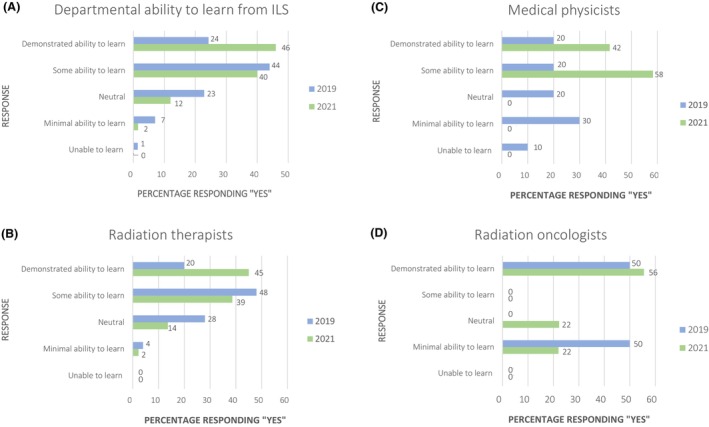
Staff reported departmental learning capacity from ILS in 2019 (blue) and 2021 (green) by professional cohort.

An increase was observed in the number of staff who thought the department demonstrated the ability to learn from the ILS, from 24% at baseline to 46% in 2021 post‐implementation of LIRON (Fig. 5A). By professional group, RTs demonstrated the strongest perception of departmental learning capacity, while ROs showed the least (Fig. 5B,D).

Surveyed staff were asked to rank their preferences for learning and feedback of the ILS. Pre‐ILS implementation, the most preferred method was an open all‐staff MDT meeting, which shifted to a selected staff meeting post‐implementation. RTs preferred a newsletter/email notification followed by all staff attendance at an MDT meeting. Learning preferences for ROMPs and ROs were similar with the highest being selected was ‘staff MDT meeting attendance’. Selected attending staff were expected to give feedback to their wider professional group at staff meetings.

## Discussion

This study of ILS engagement and SC found positive impact in the first year of experience with the LIRON ILS. The overall survey response rate within a professional group was highest for ROMPs, followed by RTs and ROs. This response rate by cohort is similar to Cunningham et al.^33^ who reported a lower response rate of 35% for physicians.

Errors can occur at any stage of the patient pathway. Incidents within the department involved in this study were most commonly detected at treatment delivery and pre‐treatment QA checkpoints, which have the highest involvement from RTs, followed by ROMPs. For the first 12 months of LIRON implementation, reporting was predominantly completed by RTs, followed by ROMPs. Reporting rates across cohorts are similar to Hartvigson et al.,^14^ Greenham et al.^26^ and Smith et al.^30^ who also found the majority of reports were submitted by RTs followed by ROMPs and ROs. Results from this study support the current literature that frontline staff are more likely to detect incidents at QA checkpoints.^26^, ^29^, ^30^

A survey by Spraker et al.^34^ found more than 60% of RO registrars had little or no exposure to incident learning systems and were not confident leading patient safety quality improvement initiatives. Further findings by Smith et al.^35^ highlight ROs are less likely to report and the errors they encounter may be of higher risk severity (eg. incorrect prescription, tumour laterality, target definition). Detection of these high‐risk errors is difficult as it requires increased specialisation and may not be obvious to other members of the MDT.

Kundu et al.^36^ suggests the low rates of reporting from ROs could be attributed to cultural factors, fear, time constraints or lack of awareness. Results of this study warrant further investigation into reporting barriers for ROs and highlight the need for education to improve engagement from this discipline.^3^ The highest proportion of incidents were related to the treatment preparation process, most being discrepancies in documentation with a high probability of being corrected before treatment. Results from this study are comparable to Yeung et al.^37^ who also found documentation accounted for the greatest proportion of incidents, with over half due to error in data transfer and the remainder related to staff miscommunication and unclear or inconsistent instructions. Lack of, or inadequate documentation, or failure to follow documentation, was also found by other authors to be a primary cause of incidents.^13^, ^29^ The report ‘Towards safer radiation therapy’ further highlighted ineffective transfer of information, poor communication and departmental hierarchy were the most common causes of errors in radiation therapy.^3^

Overall, a reduction in perceived barriers to reporting was observed. The leading barrier to reporting perceived by respondents was time taken to complete the report, which increased slightly post‐ILS implementation. This increase could be the result of a reduction in other barriers to reporting. However, findings highlight a potential area for further improvement, as a key focus when developing LIRON was building a user‐friendly platform with quick user guides to facilitate rapid data entry.

The impact of time on reporting participation could also be attributed to the inertia of completing the report.^7^ Interview findings from Hewitt and Chreim^38^ found healthcare providers don't prioritise reporting if an incident is resolved in the busy clinical environment. Instead they utilise a ‘fix and forget'approach to handling near misses not significant enough to cause actual harm to the patient and focusing on solving individual patient safety issues.

Another key feature of developing LIRON was maximising accessibility to the system. The significant decrease of this perceived barrier reflects a positive outcome of LIRON and is largely due to its integration onto the Varian Aria™ platform. These results are comparable with those reported elsewhere in radiation oncology literature.^21^, ^29^ Ford et al.^7^ noted an electronic ILS in the radiation oncology context increased reporting accessibility to strengthen engagement and facilitate event status tracking of investigation.

Respondents across all cohorts demonstrated an improved understanding of using the ILS, which may be attributed to in‐service training, mandatory new staff orientation and training initiatives to upskill and refresh all radiation oncology staff. Educational efforts regarding the purpose and importance of incident reporting aim to improve staff knowledge and use of ILS. A designated full‐time position for a quality and safety RT within the department has enabled continued awareness, operation and development of the LIRON ILS. Furthermore, continued ILS participation may be attributed to support from senior management to promptly institute quality improvements as a result of incident findings. These efforts also described by Woodhouse et al.^19^ and Yang et al.^23^ promote a normative safety culture which is visible to all staff.

RTs showed the biggest increase in ILS use and understanding while ROMPs demonstrated the least (Fig. 4). The difference in understanding and use of the ILS between cohorts highlights a gap in knowledge, which can be used to inform future training needs and engagement with the system.

Post‐ILS implementation, a reduction in perceived barriers that are considered contributors to a negative safety culture was observed. These barriers include punitive concerns or not seeing the benefit of incident reporting.

Positive SC is associated with fewer adverse events (e.g. actual incidents).^13^, ^29^ The number of respondents who believed the department did not practice no‐blame culture decreased to less than half the original percentage, implying positive safety culture increased as a result of the new ILS. ROMPs demonstrated the most significant change in perception of departmental no‐blame culture. However, this large change may be due to the initial survey only engaging 20% of ROMPs while the follow‐up engaged 100% of ROMP staff and provided a more accurate indication of their SC perceptions.

Findings from the published literature recognise the significance of fear of blame that can deter ILS participation.^13^, ^29^ Survey responses from Woodhouse et al.^21^ found individuals are disinclined to be transparent regarding their experiences of error due to an assumption they will be held responsible or punished. This is further supported by Church et al.^22^ who described the greatest barriers to reporting were fear of punishment, followed by departmental hierarchy and poor communication.

Previously, WSLHD utilised a paper reporting system alongside the older NSW Health Incident Information Management System (IIMS). Radiation oncology staff expressed that those systems did not provide feedback to users, which was a missed learning opportunity.^8^

Overall, surveyed respondents showed a preference for learning and feedback from the ILS in the form of a staff meeting. Currently, the department utilises monthly LIRON meetings hosted through Microsoft Teams™ to maximise staff attendance around clinical duties. It is open for attendance by all RTs, ROMPs and ROs to discuss actual incidents, learning opportunities and assess themes in data trends. Email distribution of monthly LIRON minutes and summary of submitted reports provide feedback to all staff within the department.

The RT representative presents at monthly radiation oncology quality assurance morbidity and mortality (RO QA M&M) meetings to share learnings and quality improvement (QI) changes that have been recommended or commenced from LIRON meetings. If there is disagreement, this is taken back to LIRON meetings for further follow‐up discussion. This meeting structure was found to best align with the operational structure of WSLHD.

The introduction of the LIRON ILS has been critical in evaluating the effectiveness of departmental checking procedures in error detection. However, errors, particularly human derived, cannot be completely prevented. It is unlikely that the ILS could be improved just by increasing QA checking points; instead, it may overwhelm the system and reduce efficiency. Radicchi et al.^11^ suggests a more effective approach to be ‘closing the safety feedback loop’, which describes implementation of corrective actions for process improvement and dissemination of lessons learnt to raise safety awareness. Effective feedback from the ILS assures staff reporting is worthwhile as reports are acknowledged, investigated and acted upon rather than falling into an administrative ‘black hole’. Survey findings from Woodhouse et al.^21^ found RTs were more likely to participate in reporting if their suggestions about patient safety were acknowledged.

The LIRON ILS has provided valuable guidance to identify clinical areas or workflow processes that would benefit from quality and safety improvements. Data from incident reporting have been useful to evaluate corrective measures and recognise ineffective processes. Reporters are encouraged to provide recommendations for future prevention when completing LIRON reports. Learning actions as a result of LIRON implementation include focused education in‐services/sessions, changes in protocols/guidelines and quality control measures for continuous improvement.

Strengths of this study include similar overall response rates pre‐ and post‐ILS implementation; representation from each professional cohort and anonymisation likely contributed to a higher response rate. However, there are a number of limitations to the study. Although findings are consistent with radiation oncology literature, these findings are from a single institution and may not be generalisable to other departments or geographies. The inclusion of ‘sometimes’ as a response option at the follow‐up survey, which was previously written by multiple staff in the open text box does not allow a completely like‐for‐like comparison.

Potential bias may have occurred due to the voluntary nature of the survey, it is possible that staff with a positive view of SC and greater understanding of incident learning were more likely to complete the survey and positively skew findings. Additionally, survey fatigue may have impacted engagement of the post‐ILS implementation survey as it was actively open at the same time as two other surveys on different topics within the hospital.

Furthermore, implementation of the LIRON ILS and the post‐implementation survey was completed during the COVID‐19 pandemic where staffing and resources were significantly reduced. At the time of the 12‐month post‐implementation survey, the department was operating at ‘red high alert’ due to increased government restrictions. The added stressors on staff from additional personal protection equipment (PPE), changes in normal workflow and hybrid working from home due to COVID‐19 may have reduced survey engagement.

## Conclusion

Overall, the introduction of LIRON was shown to have had a positive impact on reporting and safety culture within the department, while also addressing multiple issues identified in the previous manual (paper‐based) reporting system. Respondents indicated reduced barriers to reporting and increased education, feedback and MDT collaboration.

Survey findings correlate with the literature from other countries suggesting ILSs and safety culture have a positive impact on patient safety. Our department has recognised the benefit of involving all staff, regardless of seniority, in QI and safety initiatives to maintain a robust safety culture and continual learning. Findings from the first 12 months of LIRON utilisation are positive. However, continued assessment to determine its impact long‐term is recommended.

The ILS and safety culture survey has been repeated late 2023, which is 3‐year post‐implementation. It is anticipated the survey will be repeated every 3–5 years to monitor staff perceptions, identify areas of improvement and ensure a just safety culture is maintained. This time frame permits sufficient time to assess the impact of the ILS as well as future upgrades to the system. Future work will include the launch of LIRON 2.0, which will feature increased data capture and analysis capabilities.

## Conflict of Interest

The authors declare no conflict of interest.

## Funding Information

No funding supported this project.

## Supporting information


Appendix S1.

## Data Availability

The data that support the findings of this study are available on request from the corresponding author. The data are not publicly available due to privacy or ethical restrictions.
