# Supplementary material for: Learning in radiation oncology: 12‐month experience with a new incident learning system
Source: J Med Radiat Sci. 2024 Sep 15;72(1):63–73. doi: 10.1002/jmrs.823 (PMC11909703; doi:10.1002/jmrs.823)
Supplement: Supplementary file 1 — Appendix S1. [file JMRS-72-63-s001.docx]

**Appendix 1**

| **Q1. Are you aware of any incident reporting or learning systems within your Radiation Oncology Department?** |
| --- |
| Yes |
| No |
| **Q2. How many incident reporting systems are you aware of that can be used by your department?** Eg. RT specific, hospital wide, statewide. |
| 1 |
| 2 |
| 3 |
| 4 |
| 5 |
| Don’t know |
| **Q3. Please state the name of any reporting system/s you know of used by your department.** |
| Open text response box |
| **Q4. Have you submitted an actual, near miss incident or procedural non-compliance report in the last 12 months?** |
| Yes |
| No |
| **Q5. What is the reason you did not submit a report in the last 12 months?** |
| Choose not to report |
| Did not notice, observe or discover any incident or near miss event or procedural non-compliance in the past 12 months. |
| Informed team leader who investigated and submitted the report |
| Unsure how to complete a report |
| Requested another staff member to complete report on my behalf |
| **Q6. Please rate how encouraged you feel to report actual, near miss incidents or procedural non-compliance within your radiation therapy department?** |
| Very Discouraged to report |
| Discouraged to report |
| Neutral |
| Encouraged to report |
| Very Encouraged to report |
| **Q7. Do you feel comfortable reporting actual, near miss incidents or procedural non-compliance within your radiation therapy department?** |
| Very uncomfortable reporting |
| Uncomfortable reporting |
| Neither comfortable or uncomfortable |
| Comfortable reporting errors |
| Very comfortable reporting errors |
| **Q8. Do you feel that your radiation oncology department practices a culture of no-blame when errors are reported?** |
| Yes |
| No |
| Sometimes |
| **Q9. Have you ever personally received or witnessed other staff members receiving negative action towards them due to a reported actual incident, near miss incident or procedural non-compliance?** |
| Yes |
| No |
| Do not wish to answer |
| **Q10. What do you find is the biggest obstacle to you reporting actual incidents, near miss radiation incidents or procedural non-compliance in your department?** (tick all that apply) |
| Takes too long |
| System is hard to access |
| Don’t know how to use/or understand the system |
| Don’t see the benefit of reporting |
| Fear of negative action towards self or other staff |
| I do not think there are any obstacles to reporting in my department |
| **Q11. In your opinion after an actual incident, near miss incident report or procedural non-compliance is submitted the cause/blame is mostly assigned to:** |
| 0% on staff member, 100% on current process/resources |
| 25% on staff member, 75 % on current process/resources |
| 50% on staff member, 50 % on current process/resources |
| 75% on staff member, 25 % on current process/resources |
| 100% on staff member, 0 % on current process/resources |
| **Q12. To whom do you speak to first once you identify an actual incident?** |
| Staff involved in the incident |
| Senior Radiation Therapist |
| Team Leader |
| HOT/HOP |
| Director of Radiation Therapy |
| **Q13. To whom do you speak to first once you identify a near miss incident?** |
| Staff involved in the incident |
| Senior Radiation Therapist |
| Team Leader |
| HOT/HOP |
| Director of Radiation Therapy |
| **Q14. To whom do you speak to first once you identify a procedural non-compliance?** |
| Staff involved in the incident |
| Senior Radiation Therapist |
| Team Leader |
| HOT/HOP |
| Director of Radiation Therapy |

| **Q15. Do you feel that you can correctly identify when a radiation incident is an actual incident, near miss incident or procedural non-compliance'?** |
| --- |
| All of the time |
| Some of the time |
| Not at all |
| Situational dependent |
| **Q16. Do you feel that you can correctly identify the error type in relation to the 9 national practice standards?**  1-Prescription Related, 2-Simulation Related, 3-Computer Planning Related, 4-Pre-Treatment Related, 5-Treatment Related, 6-Bolus Related, 7-Shielding / MLC Related, 8-Verification Imaging Related, 9-Documentation Related. |
| All of the time |
| Some of the time |
| Not at all |
| Situational dependent |
| **Q17. How well do you believe your department is willing and able to learn from the incident learning system?**  Such as making positive process changes and or implementing appropriate training and education when necessary. |
| Unable to learn from previous incidents. |
| Minimal ability to learn |
| Neutral |
| Some demonstrated ability to learn |
| Demonstrated ability to learn from previous incidents. |
| **Q18. What methods does your department use to provide learning and feedback from reported actual incidents, near miss radiation incidents or procedural non-compliance?**  (tick all that apply) |
| All staff attendance at MDT incident reporting meeting |
| Selected staff attendance at an incident reporting meeting ( e.g., Team leaders, or Safety/Quality team) |
| Attending in-service training |
| Newsletter or email notification |
| Word of mouth |
| None |
| Other |
| **Q19. Rank your preference for feedback and learning from reported actual and near miss radiation incidents.**  Drag into position order, or use arrows that appear when you hover on the right of the bar. Rank: 1 being most preferred and 6 being least preferred. |
| All staff attendance at MDT incident reporting meeting |
| Selected staff attendance at an incident reporting meeting ( e.g., Team leaders, or Safety/Quality team) |
| Attending in-service training |
| Newsletter or email notification |
| Word of mouth |
| None |
| **Q20. Who performs any formal investigation of a reported actual, near miss radiation incident or procedural non-compliance?**  Tick all that apply. If other, select box and please only use stated role (e.g. QA RT). Do not provide individual staff names. |
| Radiation Oncologist |
| Radiation Therapist |
| Radiation Oncology Medical Physicist |
| Not sure who investigates reports |
| Other |
| **Q21. What is your role?** |
| Radiation Therapist General Base Grade |
| Radiation Therapist Specialist E.g.: Grade 3, Site specialist, Advanced Practitioner, Educator, Researcher |
| Radiation Therapist Team Leader/ Senior |
| Radiation Therapist Management [HOT/P, Deputy/Director] |
| Radiation Oncology Medical Physicist: Registrar |
| Radiation Oncology Medical Physicist: Specialist |
| Radiation Oncology Medical Physicist: Senior or above |
| Radiation Oncologist: Registrar |
| Radiation Oncologist: Fellow, Staff Specialist |
| Other |
| **Q22. How many years qualified are you in your profession?** |
| 0-2 |
| 3-5 |
| 5-10 |
| 10-20 |
| 20+ |
